# CAR T cells for HLA desensitization: a new era in kidney transplantation

**DOI:** 10.1038/s41392-026-02989-7

**Published:** 2026-09-30

**Authors:** Peter R. Mertens, Eugen Feist, Dimitrios Mougiakakos

**Affiliations:** 1 Department of Nephrology and Hypertension, Diabetology and Endocrinology, Otto von Guericke University Magdeburg, Magdeburg, Germany; 2 Magdeburg Center for Cell- and Immunotherapy, Otto von Guericke University Magdeburg, Magdeburg, Germany; 3 Department of Experimental Rheumatology, Otto von Guericke University, Magdeburg, Germany; 4 Department of Hematology, Oncology, Cell- and Radiotherapy, Otto von Guericke University Magdeburg, Magdeburg, Germany

**Keywords:** Transplant immunology, Translational research

In a recent study published in the *New England Journal of Medicine*, autologous CD19- and BCMA-directed chimeric antigen receptor (CAR) T-cell therapy was evaluated as a desensitization strategy in two highly sensitized kidney transplant candidates.^1^ In this safety run-in cohort of an ongoing phase I clinical trial (NCT06056102), CAR T-cells reduced anti-human leukocyte antigen (HLA) antibody levels, enabling transplantation and providing the first clinical proof-of-concept for CAR T-cell-based HLA desensitization.

Anti-HLA antibodies remain a major obstacle to kidney transplantation. Sensitization is quantified using the calculated panel reactive antibody (cPRA) score, to estimate the percentage of potential organ donors expected to be incompatible (Fig. 1a). Increasing scores reduce the probability of receiving a compatible organ. The consequences can become striking: a patient with a cPRA of 99.998% is expected to be compatible with approximately one in 50,000 donors. Highly sensitized patients, especially against common HLA alleles, face substantial wait-list mortality.

**Fig. 1:**
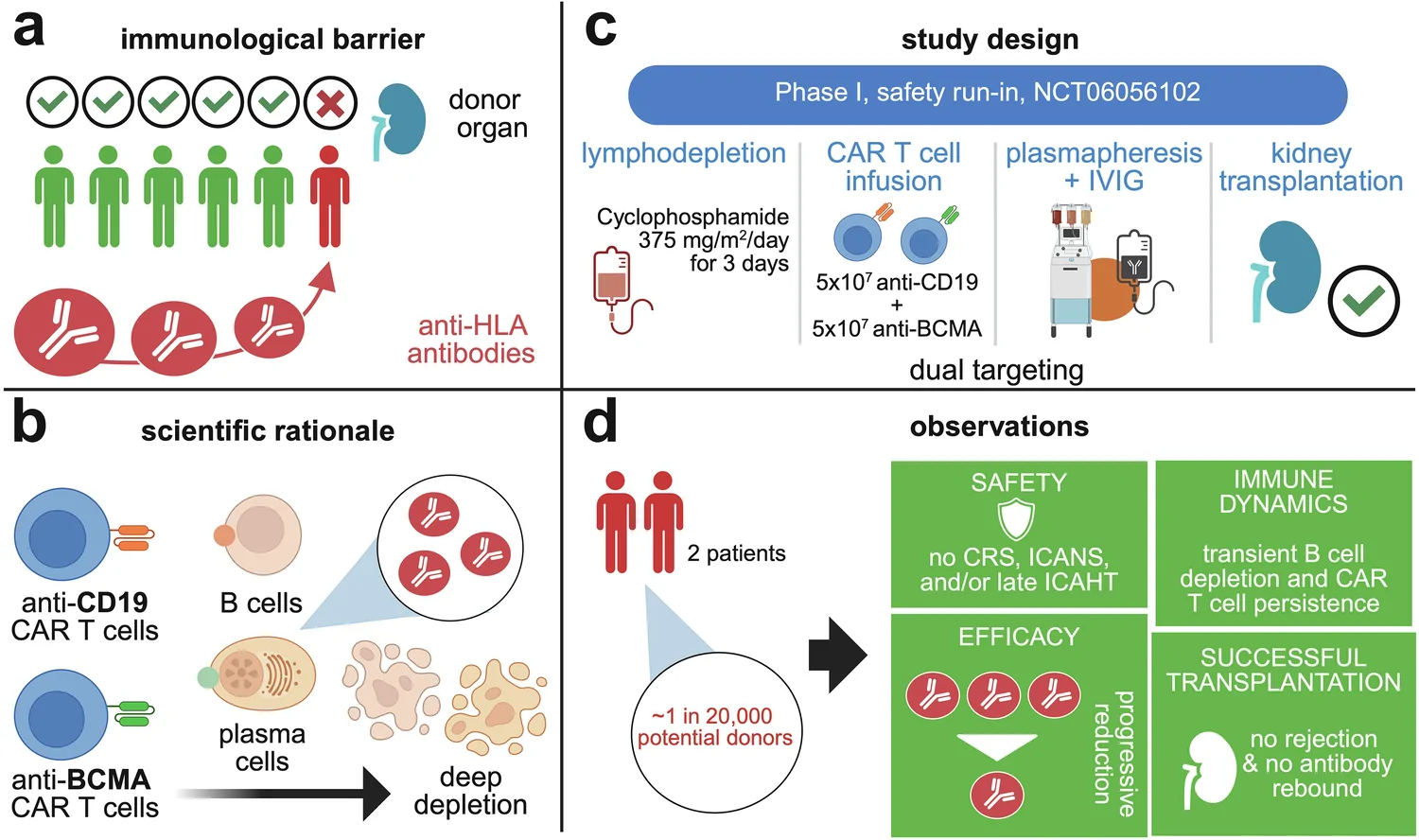
CAR T-cell therapy as a novel desensitization strategy for highly sensitized kidney transplant candidates. **a** Preformed anti-human leukocyte antigen (HLA) antibodies represent a major immunological barrier to kidney transplantation by markedly reducing the probability of identifying a compatible donor. **b** Dual targeting of CD19-expressing B cells and BCMA-expressing plasma cells with autologous CAR T cells aims to induce profound depletion of the cellular source of anti-HLA antibodies. **c** Following cyclophosphamide-based lymphodepletion, patients received infusion of autologous CD19- and BCMA-directed CAR T cells. Approximately one month later, plasmapheresis and low-dose IVIG were administered. The overall treatment strategy was designed to achieve sufficient desensitization to enable successful kidney transplantation. **d** In the safety run-in cohort, two highly sensitized kidney transplant candidates were treated. CAR T-cell therapy was well tolerated, induced transient B-cell aplasia with limited CAR T-cell persistence, substantially reduced anti-HLA antibody levels, and enabled successful kidney transplantation without donor-specific antibody rebound or evidence of rejection during 7–12 months of post-transplant follow-up. BCMA B-cell maturation antigen, CRS cytokine release syndrome, ICANS immune effector cell-associated neurotoxicity syndrome, ICAHT immune effector cell-associated hematotoxicity, IVIG intravenous immunoglobulin

Current strategies to facilitate transplantation follow two principles. The first seeks to increase access to organs through optimized allocation systems and kidney paired donation programs. The second aims to reduce alloantibody levels by desensitization. Various approaches are utilized, including plasmapheresis combined with intravenous immunoglobulin (IVIG), B-cell depletion using monoclonal antibodies or IgG-cleaving enzymes. However, sustained responses remain uncommon most likely because the underlying antibody-producing cells persist, leading to a so-called antibody rebound and risk of rejection. Consequently, durable desensitization remains a major unmet clinical need.

The rationale for employing CAR T-cell therapy in this setting originates from its recent success in autoimmune diseases. Since the first report of CD19-directed CAR T-cells inducing long-lasting remission in refractory systemic lupus erythematosus, the field has rapidly expanded.^2^ CAR T cells induce deep B cell depletion, thereby eliminating potential pathogenic populations. Interestingly, many patients maintain treatment-free remission despite B-cell reconstitution, suggesting durable restoration of immune tolerance through an “immune reset.” While many questions remain, including optimal target antigens, current clinical experience suggests that CAR T-cell therapy in non-malignant diseases has a more favorable safety profile, likely reflecting the lower antigen burden.^3^

Building on this concept, the authors investigated whether deep depletion of anti-HLA antibody-producing cells could reshape humoral alloimmunity sufficiently to facilitate transplantation in highly sensitized patients and thereby enable transplantation. To maximize depletion across B-cell and plasma-cell compartments, they employed a dual-targeting strategy using separate autologous CD19- and B-cell maturation antigen (BCMA-)directed CAR T-cell products (Fig. 1b).

As part of an early clinical evaluation for highly sensitized kidney transplant candidates with a cPRA score of ≥99.5%, two 54- and 47-year-old male patients received a relatively low dose of 5 × 10^7^ CD19-directed and 5 × 10^7^ BCMA-directed CAR T cells following a lymphodepleting regimen consisting of cyclophosphamide (375 mg/m² for 3 consecutive days) without fludarabine. Beginning one month after CAR T-cell infusion, both patients underwent five sessions of plasmapheresis, each followed by IVIG to accelerate clearance of alloantibodies (Fig. 1c). Both had previously undergone two kidney transplantations, had lost their most recent kidney allograft due to antibody-mediated rejection, and presented with baseline cPRA values of 99.998% and 99.993%, respectively.

Treatment was well tolerated, with no high-grade cytokine release syndrome or neurotoxicity. Hematologic toxicities were transient; no prolonged or late cytopenias occurred, and the immunological response closely resembled that previously observed in patients with autoimmune diseases. Both CD19- and BCMA-directed CAR T-cell products underwent robust in vivo expansion, resulting in profound depletion of circulating B cells. CAR T-cell persistence and the associated B-cell aplasia lasted approximately three to 4 months, followed by B-cell reconstitution. The recovering B-cell compartment consisted predominantly of naïve and transitional B cells, suggesting remodeling of the B-cell compartment; however, whether this translates into durable reshaping of HLA-specific immune memory remains unknown. Notably, patient 2 developed BK viremia after transplantation, a complication that is well recognized in the context of post-transplant immunosuppression. However, larger studies are required to determine the extent to which the combination of CAR T cell-mediated B- and plasma-cell depletion and conventional post-transplant immunosuppression increases the cumulative risk of infections. This question is of particular importance, as infectious complications remain the leading causes of treatment-related mortality following CAR T-cell therapy in patients with malignancies (Fig. 1d).

Additional evidence of deep tissue depletion was obtained in patient 1, in whom fine-needle aspiration of an axillary lymph node performed 2 months after CAR T-cell infusion demonstrated complete absence of B cells. Likewise, bone marrow analyses in both patients revealed profound plasma-cell depletion at month 2. Interestingly, the plasma-cell phenotype differed between the two patients: whereas plasma cells in patient 1 were predominantly CD19-positive at baseline, they were largely CD19-negative in patient 2. Importantly, these observations relate to the total bone marrow plasma-cell compartment. Whether pathogenic alloantibody-producing plasma cells exhibit similar phenotypic heterogeneity remains unknown. Nevertheless, they suggest that plasma-cell biology may differ between patients and raises the possibility that the optimal therapeutic target, i.e., CD19, BCMA, or a combination of both, may differ between individual patients.

Most importantly, both patients experienced progressive reductions in anti-HLA antibody levels, allowing delisting of unacceptable HLA antigens and reductions in cPRA scores. Patient 1 showed a reduction in cPRA from 99.998% to 99.516% and underwent successful kidney transplantation on day 229 after CAR T-cell infusion. Similarly, patient 2 achieved a reduction in cPRA from 99.995% to 99.567% and received a kidney transplant on day 93. During follow-up, both patients maintained stable graft function without evidence of antibody-mediated rejection or rebound of donor-specific antibodies (Fig. 1d).

Overall, these findings represent promising preliminary data and are supported by a study in which CD19-directed CAR T cells successfully eliminated anti-HLA antibodies.^4^ This observation raises the question: is dual CD19- and BCMA-targeting required, or may CD19-directed therapy alone be sufficient? At present, biomarkers to guide this choice are lacking, while emerging data suggest that BCMA-directed therapies may induce broader B-cell depletion than initially anticipated. Moreover, the current findings were generated using intentionally reduced CAR T-cell doses in a safety run-in cohort. Whether higher dose levels will achieve more profound depletion of pathogenic alloantibody-producing cells while maintaining an acceptable safety profile remains an important question that is currently being addressed. Future studies should also define how breadth of HLA sensitization (i.e., cPRA score) together with antibody characteristics, including antibody strength and serum dilution analyses, can guide the selection of the most appropriate desensitization strategy. As in autoimmune diseases, CAR T-cells will also need to be benchmarked against T-cell engagers. These agents redirect polyclonal T cells against target antigens without genetic modification. Recent data with BCMA-targeted T-cell engager already indicate their potential.^5^ These findings may mark the beginning of a new era of desensitization in transplantation.

## References

[1] Bhoj, V. G. et al. Kidney transplantation in two highly sensitized candidates after CAR T-cell therapy. *N Engl J Med* **394**, 2117–2125 (2026). 10.1056/NEJMoa2513428 PMC13240644 42235014

[2] Mougiakakos, D. et al. CD19-targeted CAR T cells in refractory systemic lupus erythematosus. *N Engl J Med* **385**, 567–569 (2021). 10.1056/NEJMc2107725 34347960

[3] Mougiakakos, D., Meyer, E. H. & Schett, G. CAR T cells in autoimmunity: game changer or stepping stone? *Blood* **145**, 1841–1849 (2025). 10.1182/blood.2024025413 PMC12782971 39700499

[4] Schrezenmeier, J. et al. Kidney Transplantation after clearing anti-HLA antibodies with CD19 CAR T cells. *N Engl J Med* **394**, 2166–2168 (2026). 10.1056/NEJMc2517277 42235019

[5] Leon, J. et al. B-cell maturation antigen-Targeted T-cell engager therapy combined with B-cell depletion for treatment of refractory HLA sensitization. *Kidney Int* **109**, 1148–1154 (2026). 10.1016/j.kint.2026.01.020 41692345

